# Gamification in Stomatherapy: Virtual Game for Pressure Injury Care

**DOI:** 10.1155/ijta/4526690

**Published:** 2026-01-08

**Authors:** Kamila Mendes Malheiros, Lucas Gonçalves Alves, Rosa Maria Esteves Moreira da Costa, Helena Maria Scherlowski Leal David, Hermes Cândido de Paula, Donizete Vago Daher, Carolina Neves Dias de Andrade, Vera Maria Benjamim Werneck, Magda Guimarães de Araujo Faria

**Affiliations:** ^1^ Núcleo de Telessaúde e Saúde Digital, Universidade do Estado do Rio de Janeiro, Rio de Janeiro, Brazil, uerj.br; ^2^ Divisão de Ações e Programas de Saúde, Secretaria Municipal de Saúde do Rio de Janeiro, Rio de Janeiro, Brazil; ^3^ Escola de Enfermagem Aurora de Afonso Costa, Universidade Federal Fluminense, Niterói, Brazil, uff.br; ^4^ Faculdade de Enfermagem, Universidade do Estado do Rio de Janeiro, Rio de Janeiro, Brazil, uerj.br

**Keywords:** enterostomal therapy, gamification, nursing, pressure ulcer, professional training

## Abstract

**Introduction:**

Pressure injuries are injuries caused by reduced blood circulation over regular and repetitive periods when applying pressure to a specific area of the body. This incident requires daily prevention and health promotion to prevent its occurrence.

**Objective:**

To develop and evaluate a virtual board game with guidelines for pressure injury prevention for nurses specializing in stoma therapy.

**Methodology:**

This is an applied, exploratory, and descriptive technological development study. The game was developed in stages consisting of: conceptual review, definition of the game dynamics, initial design of the board and cards, creation of the cards, and definition of the components. The study stages include development with a specialist team, testing and validation of the platform with specialist nurses, and subsequently, release of the final product.

**Results:**

The results showed high acceptance of the game among the 17 participants, with emphasis on high levels of engagement and learning (≥ 94% reported acquiring new knowledge), ease of use (≥ 82% strongly agreed with the intuitiveness of the interface), and intention to continue using it (94% expressed a desire to continue using and recommend the tool). The main limitations identified were related to technical difficulties during installation and the lack of interactive features for multiple users.

**Conclusion:**

The results of this study reinforce the potential of gamification as a complementary and innovative strategy for teaching stomatherapy. The prototype proved to be an effective and well‐accepted tool for educating nurses on pressure injury prevention, with significant potential for improving knowledge and clinical practice. It is believed that gamification, combined with theoretical teaching and practical learning, can mobilize and engage participants, significantly contributing to the development of highly qualified professionals and, consequently, improving the quality of patient care.

## 1. Introduction

Pressure injury (PI) is defined as localized damage to the skin and/or underlying soft tissues, typically occurring over a bony prominence or in relation to the use of medical devices or other artifacts. It may manifest as an injury on intact skin or as an open wound, with or without pain, and can largely be prevented with appropriate management [1, 2].

PIs are a public health issue, highly complex and multifactorial in nature. The occurrence of these injuries causes physical and emotional distress to the patient, increasing the risk of complications and influencing morbidity and mortality. Furthermore, the consequences impact family members and increase the treatment costs for the healthcare system [3]. These are complex and often preventable injuries, making it essential that health professionals, especially stoma care nurses, are able to act in prevention, classification, monitoring, and care [4].

In Brazil, the stomatherapy nurse is a professional graduated in Nursing and qualified as a specialist in stomatherapy through certified courses with at least 360 h, of which at least 20% are practical, possessing the knowledge and skills to care for people with stomas, wounds, fistulas, catheters, drains and anal, and urinary incontinence [5]. This nurse is responsible for participating in the assessment, development of protocols, selection, indication and prescription of dressings, and adjuvant technologies to promote care, as well as the prevention, treatment and rehabilitation of people with skin lesions, including health education actions aimed at the affected user and their families [6].

The development of gamified digital strategies to improve the skills of nurses working in this area is recognized in the scientific literature as a viable and effective strategy, as it stimulates participant engagement and overcomes limitations related to time and possible travel [7, 8].

On the other hand, clear limitations have been identified in the use of digital technologies in stomatherapy education, such as students′ difficulty in connecting theoretical teaching with nursing practice, which further increases the relevance of engaging and gamified strategies, as these induce the simulation of real situations that help in understanding the care to be performed [7, 9].

In general, the use of gamification in nursing is a relevant strategy for developing skills and competencies such as critical thinking about one′s own practice, therapeutic decision‐making, and, in some cases, even team communication [10]. Although research addressing the effectiveness of gamification in stomatherapy is scarce, a recent study conducted in Turkey showed that nursing students who used gamified strategies demonstrated greater ability to provide care to patients with stomas than students who used traditional strategies [7].

In nonspecific fields of nursing, gamification has been observed to positively influence student motivation and confidence, as it stimulates cognitive, motivational, and behavioral outcomes [11]. Therefore, its complementary use should be encouraged, especially in continuing education settings [12, 13].

This study is aimed at developing and evaluating a gamified virtual board game to guide PI prevention among nurses specializing in enterostomal therapy. The proposal is justified by the need for innovative strategies that engage and enhance competencies in PI prevention and care, promoting patient safety and strengthening nursing education. The gamified virtual board game was chosen for its intuitive dynamics, high engagement potential, immediate feedback, active and scenario‐based learning, the possibility of safe repetition of practices, personalized pacing, remote accessibility, and performance measurement.

## 2. Materials and Methods

This is a convergent care research, which is a method that seeks to combine factors present in clinical practice with research actions in the healthcare field. It is conducted by a researcher with expertise in the area of knowledge addressed in the study. In other words, it is a methodology that aims to integrate improvements and innovations into clinical practice based on problems arising from professional practice [14, 15].

Thus, when using this methodology, it is necessary for there to be convergence between research actions and clinical practice actions, with an overlap between research and care [15].

In this investigation, the technological development was focused on the creation of a virtual board game with guidance for the prevention of pressure injuries for nurses. The following question served as the foundation: How would the ideal game for supporting the learning of stomatherapy nurses, focusing on PI prevention, look?

### 2.1. Prototype Creation

The game was developed collaboratively by a team consisting of specialized nurses, professionals from computer science, and software developers. The development of the game was carried out using the Unity tool, Version 2022.3.4f. The code was written in the C# programming language, and the graphics were designed using the Figma tool. After the prototype was developed, usability tests were conducted by the research participants, with appropriate adjustments made to align with the proposed objectives.

The game′s content was entirely developed by the project team, based on international manuals and protocols for the care of people with stomas, as well as bibliographic content obtained through a literature review. The cases presented were entirely captured in the researchers′ clinical practice, and the images can be found in public databases available online.

### 2.2. Prototype Formative Evaluation

The evaluation was based on the game′s effectiveness criteria and its achievement of its proposed objectives. The criteria were divided into four phases (1) the games ability to engage the user in learning about PI prevention; (2) ease of operation; (3) frequency of use; (4) satisfaction with use and results obtained.

The participants were nurses specializing in stoma therapy and nurses in training in this specialty. This profile is justified because, in Brazil, PI care is performed exclusively by stoma therapists. Eighteen professionals were recruited from a specialization course accredited by the Brazilian Association of Stoma Therapy. One participant was excluded for having completed the assessment without actually accessing the game; therefore, the sample consisted of 17 participants. Data collection took place virtually between October and November 2023 and consisted of providing the game, sending installation instructions, and administering an online questionnaire for data collection.

Participants were recruited through advertising in specialization classes, where the prototype was presented and its use encouraged over the course of 3 weeks. Use occurred both in the classroom, during regular activities, and at other times, including continuing education activities conducted at home. Participants were not given instructions on how often to use the game.

The data collection instrument consisted of 27 questions, responses to which should be indicated on the following scale: 1, *disagree*; 2, *partially disagree*; 3, *neither disagree nor agree*; 4, *partially agree*; 5, *agree*. The lower the score, the lesser the participant agreed with the statement, and conversely, the higher the score, the more they agreed. Furthermore, an open‐ended question was incorporated into the questionnaire, allowing participants to suggest suggestions for the game. The analysis was based on descriptive statistics.

### 2.3. Ethical Considerations

The research was submitted to the ethics committee of the State University of Rio de Janeiro, meeting all the legal requirements in force and being approved under protocol n° 6.338.657. For data collection, the research followed all procedures provided for by current ethical guidelines. Data collection occurred after informed consent from participants, where respect for human dignity, anonymity, confidentiality of data, provision of information on the risks and benefits of the research, and the right to withdraw from participation at any time were guaranteed.

## 3. Results

The development of the educational game followed these phases: defining the game dynamics, designing the initial board, creating cards, and defining the components and decision variables to be represented in the game. Subsequently, the prototype evaluation was carried out.

### 3.1. Development of the Prototype Dynamics

The game is a virtual board game where the user starts at the starting point and must reach the final point by answering questions about PI prevention along the way, interacting with the existing cards. During the journey, the user may land on spaces that, based on their specific dynamics, cause the user to move forward or backward on the board. The game can be played individually. The user is represented on the board by a nurse icon.

**Figure 1:**
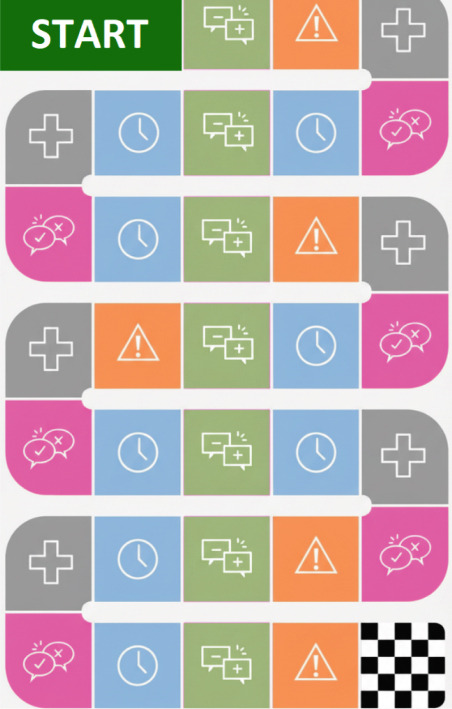
Board in vertical layout.

To adapt the game for tablets and smartphones, the game will be displayed in a vertical layout, as shown in Figure 1 below. The board consists of spaces that represent the cards, each depicted by a symbol, and at random points, it presents reference points from the daily practice of the nursing team, represented by the nursing station. The user moves around the board according to the roll of a virtual die that counts from 1 to 6, meaning they can move up to six spaces at once.

### 3.2. Creation of Cards and Definition of Components and Decision Variables

Along the board, symbols are provided that represent their respective cards, including the possibility of true or false cards, care time, PI detected, and luck or setback cards.

**Figure 2:**
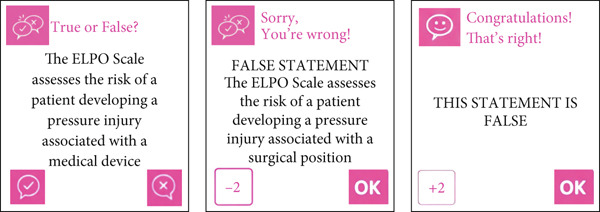
Example of a true or false card.

The “true or false” cards present a statement to the user, who must confirm whether it is true or false. If correct, the player advances to random spaces. If incorrect, the player moves backward randomly. When the statement is false, a card with the correct answer appears for the player. Twenty cards were created in this category. An example of the true or false card is shown in Figure 2.

**Figure 3:**
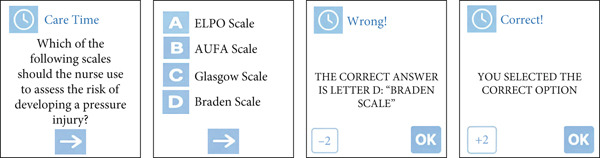
Example of a care time card.

The “care time” cards consist of questions that present four alternatives, with one correct answer. If the player selects the correct alternative, they advance spaces; if they choose incorrectly, they move backward. When the player selects the wrong option, a message with the correct answer appears on the screen. Eight cards were created in this category. An example of the care time card is shown in Figure 3.

**Figure 4:**
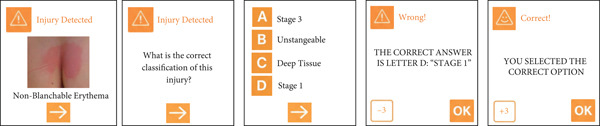
Example of a pressure injury detected card.

The “pressure injury detected” cards feature an image of a PI that appears on the screen, and the player must select the classification of that injury. If correct, the player advances to a random number of spaces; if incorrect, they move backward. Twenty cards were created in this category. An example of the PI detected card and its possible consequences is shown in Figure 4.

**Figure 5:**
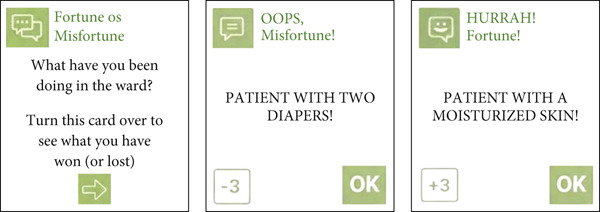
Example of a fortune or misfortune card.

In the “Fortune or Misfortune” cards, the player can receive a bonus (luck) or a penalty (setback). Each category contains 20 cards, totaling 40 cards. The luck cards contain appropriate care for the prevention of pressure injuries, whereas the setback cards feature care practices or habits from the daily nursing routine that hinder the process of injury prevention. Upon receiving a fortune card, the player can advance spaces, whereas with a misfortune card, the player may move backward. An example of a fortune or misfortune card is shown in Figure 5.

After the development of the game, functionality testing was conducted on mobile devices and Windows before proceeding to the evaluation stage. It is worth emphasizing that the product was duly registered in the national bank of technical and technological production, generating the authenticity of the material and guaranteeing the authors the right to reproduce the material for educational purposes.

### 3.3. Prototype Evaluation Results

Of the research participants, 76.5% were women (13), and 23.5% were men (4), aligning with the nursing profession′s predominantly female profile. Regarding age, 35.3% (6) were between 31 assnd 40 years old, 29.4% (5) were over 40 years old, 23.5% (4) were between 25 and 30 years old, and 11.8% (2) were under 25 years old. Regarding race, 52.9% (9) identified as White, 29.4% (5) as Brown, and 17.6% (3) as Black. Additionally, 35.3% (6) were students in the nursing stomatherapy specialization course, and 64.7% (11) were already stomatherapy nurses. The results regarding the participants′ evaluation of the game can be seen in Table 1.

**Table 1:** Prototype evaluation.

	**I disagree**	**I partially disagree**	**I neither disagree nor agree**	**I partially agree**	**I agree**
Phase 1: game′s ability to engage the user in learning about pressure injury prevention					
I can understand the objective of the game.	0	0	0	0	17
The game is useful for learning about pressure injury prevention.	0	0	0	1	16
The game is useful for discovering new knowledge about pressure injury.	0	0	0	0	17
The game provides relevant information about pressure injury prevention.	0	0	0	0	17
The game meets my need for knowledge about pressure injury prevention.	0	0	0	3	14
The game manages to maintain interest in the content it presents.	0	0	0	0	17
The game manages to stimulate the user′s initiative to study about pressure injury.	0	0	0	1	16
Phase 2: ease of game operation					
The game is easy to use as a whole.	0	0	0	3	14
The interface is intuitive.	0	0	0	2	15
I find it easy to play the game.	0	0	0	2	15
I find it easy to access information about the knowledge covered.	0	0	0	1	16
I find the game′s performance on my device satisfactory.	0	0	1	3	13
I find the game to be stable on my device.	0	0	1	2	14
Phase 3: frequency of game use					
Using a game about pressure injury prevention is interesting.	0	0	0	0	17
Using gamification to learn about pressure injuries is fun.	0	0	0	1	16
I would like to continue using the game about pressure injury prevention.	0	0	0	1	16
I would like to continue using a game that provides information about pressure injury prevention in a playful way.	0	0	0	0	17
My intention is to continue using the game to learn about pressure injury prevention.	0	0	0	2	15
I intend to recommend the game to others.	0	0	0	2	15
I intend to follow the game′s development, if it continues.	0	0	0	1	16
I intend to play with others.	0	0	1	2	14
I intend to use the game to share the proposed knowledge with others.	0	0	0	1	16
Phase 4: satisfaction with the use and results obtained with the game					
I′ve used games in nursing before.	7	3	0	3	4
I′ve used games about pressure injury prevention before.	13	1	1	0	2
My experience with the game was satisfactory.	0	0	1	2	14
I was able to learn new things with this game.	0	0	0	1	16
I believe the game has potential to be used as an educational tool.	0	0	1	0	16

The results reveal a largely positive acceptance of the prototype. In Phase 1 (engagement), there was complete agreement on items such as “understanding the game′s objective,” “discovering new knowledge,” and “maintaining interest,” with 100% of responses falling into the “agree” or “completely agree” categories. Only the item related to the “need for knowledge about prevention” received 17.6% (3) “partially agree” responses, indicating the possibility of expanding additional content in the game.

In Phase 2 (ease of operation), a small dispersion was observed, especially in the items related to the game′s performance and stability on devices. Approximately 23.5% (4) of participants reported specific difficulties, corroborating the aforementioned installation and configuration limitations. Even so, over 82% gave full agreement ratings regarding ease of use, intuitive interface, and access to information.

In Phase 3 (frequency/intention to use), the percentages were significant: 100% of participants found the game interesting, and 94% expressed an intention to continue using the tool and recommend it to colleagues, reinforcing the innovative nature of the proposal for continuing education in nursing. Only in the items related to playing with other participants was there a variation, with 17.6% (3) indicating neutrality, reflecting users′ expectations for interactive and multiplayer features.

Finally, in Phase 4 (overall satisfaction), it is noteworthy that 82.3% (14) of participants considered the experience satisfactory and 94.1% (16) reported having learned new content. The most significant finding was the almost unanimous belief (94.1%) in the game′s potential as an educational tool, which supports its practical applicability.

During the qualitative evaluation, it was observed that participants took an average of 45 min to complete the matches, and the main difficulty was related to installation, since during the testing and registration phase, the game was not yet available on the Android and iOS application platforms.

Another important point is interactivity. As it is a prototype, the game was developed for individual use; however, it was noted that users expect to interact and compete with other participants.

## 4. Discussion

The inclusion of dynamic and personalized digital teaching tools is crucial for increasing the effectiveness of healthcare educational initiatives [16]. In this context, gamification in nursing emerges as an effective strategy for enhancing the teaching experience and increasing confidence in care delivery [17, 18]. Currently, nursing is the second‐largest area of gamification studies in healthcare, accounting for an impressive 22.6% of initiatives, with studies on the topic having increased during the COVID‐19 pandemic, which may indicate a trend for the coming years [19].

The results of this study demonstrate high acceptance of the virtual game prototype, with 100% of participants understanding the game′s objective, discovering new knowledge, and maintaining interest in the content. This massive adoption corroborates data from the literature that points to the potential of gamification in nursing, not only for transmitting information but also for fostering engagement, innovation, and active learning, thereby overcoming the limitations of traditional educational methods [20]. When it comes specifically to digital board games, their use in nursing education reveals high acceptability and higher levels of knowledge retention, as well as satisfaction with the teaching method [21].

Beyond student perception, these benefits are also positively acknowledged by nursing faculty, who emphasize institutional support for the use of such tools as an essential aspect for the success of these activities [22].

The educational impact perceived by participants is one of the pillars of this study. Most nurses (94.1%) reported having learned new content and expressed the intention to continue using the tool and recommend it to colleagues. These findings address a latent demand for professional development in the field of stoma therapy, as gamification initiatives remain scarce in this area. In this sense, a game developed in Canada using realistic simulation in stoma care was observed, where it was possible to identify the potential to help improve patient care techniques [23]. Perceived learning gain and engagement are widely recognized as valid preliminary indicators of educational effectiveness in digital learning environments [24].

The aspect of cooperation and interaction emerged in the evaluation regarding users′ expectations for multiplayer features. Although the prototype was developed for individual use, 17.6% of participants indicated neutrality regarding their intention to play with others, reflecting a desire for collaborative and competitive features. The literature reinforces that interactive gamification initiatives tend to yield more significant results compared with single‐player gaming experiences, owing to enhanced motivation, social stimulation, and the establishment of agreed‐upon goals among participants [25]. On the other hand, games that excessively encourage competitiveness among users tend not to have significant adoption [26, 27].

Despite the predominantly positive evaluation regarding ease of use and the intuitive interface (over 82% full agreement), some technical difficulties were identified, mainly related to the installation and stability of the game on different devices. This limitation, while common in healthcare technology initiatives, highlights the need for accessibility optimization, including availability on application platforms and configuration improvements. Other technical barriers reported in the literature, such as a lack of integration between game elements, a lack of learning objectives, and cultural differences (such as an inability to understand digital resources), were not observed among participants [28, 29].

Digital literacy among nurses currently presents a significant barrier to the creation of new educational technology initiatives, especially since the training of these professionals focuses on user care. Digital education programs for nurses and students must respect both the objective and subjective aspects related to the different age groups of these professionals [30]. Digital literacy and the use of digital technologies not only enhance professional skills but also assist in technical activities, such as recording patient data on electronic forms [31]. Overcoming these technical barriers is crucial to ensuring the tool′s widespread adoption and success in continuing education settings.

## 5. Conclusion

This study reinforces gamification as an effective, complementary, and innovative strategy for teaching enterostomal therapy. The virtual board game prototype was well accepted and supported active, scenario‐based learning with immediate feedback, fostering engagement and new knowledge in PI prevention and informing clinical decision‐making aligned with guidelines. Participants indicated ease of use, intention for continued use, and recommendation to peers, suggesting potential for incorporation into continuing education.

Identified limitations—convenience sampling, installation/stability issues, and lack of multiplayer features—indicate avenues for improvement and research, including app‐store availability, performance optimization, collaborative/multiplayer functionalities, larger and more diverse samples, expanded clinical scenarios, and evaluation of objective impacts on clinical performance. In sum, gamification acts as a catalyst for engagement and, combined with theoretical and practical teaching, shows promise for strengthening competencies in PI prevention within stomatherapy.

## Conflicts of Interest

The authors declare no conflicts of interest.

## Funding

No funding was received for this manuscript.

## Data Availability

The data sharing is not applicable to this article as no datasets were generated or analyzed during the current study.

## References

[1] Edsberg L. E. , Black J. M. , Goldberg M. , McNichol L. , Moore L. , and Sieggreen M. , Revised National Pressure Ulcer Advisory Panel Pressure Injury Staging System, Journal of Wound, Ostomy and Continence Nursing. (2016) 43, no. 6, 585–597, 10.1097/WON.0000000000000281, 2-s2.0-84991511108, 27749790. PMC5098472 27749790

[2] Teixeira A. O. , Brinati L. M. , Toledo L. V. , Silva Neto J. F. , Teixeira D. L. P. , Januário C. F. , Silva Neto L. M. , and Salgado P. O. , Factors Associated With the Incidence of Pressure Wounds in Critical Patients: A Cohort Study, Revista Brasileira de Enfermagem. (2022) 75, no. 6, 10.1590/0034-7167-2021-0267, 35766752. PMC9728825 35766752

[3] Elli C. , Novella A. , Nobili A. , Ianes A. , and Pasina L. , Factors Associated With a High-Risk Profile for Developing Pressure Injuries in Long-Term Residents of Nursing Homes, Medical Principles and Practice. (2022) 31, no. 5, 433–438, 10.1159/000527063, 36122563. 36122563 PMC9801375

[4] Bird A. , Burch J. , and Thorpe G. , The Role of the Clinical Nurse Specialist in Stoma Care: A Scoping Review, British Journal of Nursing. (2023) 32, no. 16, S6–S16, 10.12968/bjon.2023.32.16.S6, 37682771. 37682771

[5] da C. C. C. P. , Soares S. S. S. , Vieira M. L. C. , Oliveira M. D. , Pedro R. S. , Chaves U. S. B. , and Souza N. V. , Stomatherapists in the World of Work: Practicalities and Difficulties for the Professional Practice, Escola Anna Nery. (2021) 25, no. 2, e20200262, 10.1590/2177-9465-EAN-2020-0262.

[6] Conselho Federal de Enfermagem (BR) , Resolução COFEN n° 787 de 21 de agosto de 2025. Regulamenta a atuação da equipe de enfermagem na promoção, prevenção, tratamento e reabilitação de pessoas com Lesões Cutâneas, 2025, https://www.cofen.gov.br/resolucao-cofen-no-787-de-21-de-agosto-de-2025/, s.

[7] Kulakaç N. and Çilingir D. , The Effect of a Serious Game-Based Web Application on Stoma Care Education for Nursing Students: A Randomized Controlled Trial, Teaching and Learning in Nursing. (2024) 19, no. 1, e126–e132, 10.1016/j.teln.2023.10.001.

[8] Pott F. S. , Meier M. J. , Stocco J. G. D. , de P.,. F. , Roehrs H. , and Ziegelmann P. K. , Pressure Injury Prevention Measures: Overview of Systematic Reviews, Revista da Escola de Enfermagem da USP. (2023) 57, e20230039, 10.1590/1980-220x-reeusp-2023-0039en. PMC10742601 38133528

[9] Arıburnu O. and Korkmaz F. , Nursing Students′ Perceptions and Experiences in Pressure Injury Risk Assessment: A Qualitative Study, Nurse Education in Practice. (2024) 79, 104039, 10.1016/j.nepr.2024.104039, 38996581. 38996581

[10] Sanz-Martos S. , Álvarez-García C. , Álvarez-Nieto C. , López-Medina I. M. , López-Franco M. D. , Fernandez-Martinez M. E. , and Ortega-Donaire L. , Effectiveness of Gamification in Nursing Degree Education, PeerJ. (2024) 12, e17167, 10.7717/peerj.17167, 38638160. 38638160 PMC11025539

[11] Seo Y. K. , Kang C. M. , Kim K. H. , and Jeong I. S. , Effects of Gamification on Academic Motivation and Confidence of Undergraduate Nursing Students: A Systematic Review and Meta-Analysis, Nurse Education Today. (2024) 143, 106388, 10.1016/j.nedt.2024.106388, 39303410. 39303410

[12] Del Pozo-Herce P. , Tovar-Reinoso A. , García Carpintero-Blas E. , Casaux Huertas A. , Ruiz de Viñaspre-Hernández R. , Martínez-Sabater A. , Chover-Sierra E. , Rodríguez-García M. , and Juarez-Vela R. , Gamification as a Tool for Understanding Mental Disorders in Nursing Students: Qualitative Study, JMIR Nursing. (2025) 8, no. 1, e71921, 10.2196/71921, 40540428. 40540428 PMC12204240

[13] Dutra B. K. , Silveira L. M. , Bolela F. , Lenhari M. , and Stabile A. M. , Contribution of Convergent Care Research to Preventing Pneumonia Associated With Mechanical Ventilation, Revista Enfermagem UERJ. (2021) 29, 10.12957/reuerj.2021.59821.

[14] Trentini M. , Paim L. , and Silva D. M. G. V. , The Convergent Care Research Method and Its Application in Nursing Practice, Texto & Contexto—Enfermagem. (2017) 26, 10.1590/0104-07072017001450017, 2-s2.0-85040219667.

[15] Andersen B. L. , Jørnø R. L. , and Nortvig A. , Blending Adaptive Learning Technology Into Nursing Education: A Scoping Review, Contemporary Educational Technology. (2022) 14, no. 1, 10.30935/cedtech/11370.

[16] Malicki A. , Vergara F. H. , Van de Castle B. , Goyeneche P. , Mann S. , Preston Scott M. , and Whalen M. , Gamification in Nursing Education: An Integrative Literature Review, Journal of Continuing Education in Nursing. (2020) 51, no. 11, 509–515, 10.3928/00220124-20201014-07. 33104811

[17] Min A. , Min H. , and Kim S. , Effectiveness of Serious Games in Nurse Education: A Systematic Review, Nurse Education Today. (2022) 108, 10.1016/j.nedt.2021.105178, 105178, 34717098. 34717098

[18] Chang S. J. , Kim G. M. , and Kim J. A. , The Effects of Flipped Learning and Gamification on Nursing Students′ Patient Safety Education: A Mixed Method Study, Heliyon. (2024) 10, no. 8, 10.1016/j.heliyon.2024.e29538. PMC11036057 38655326

[19] Grech J. and Grech J. , Nursing Students′ Evaluation of a Gamified Public Health Educational Webinar: A Comparative Pilot Study, Nursing Open. (2021) 8, no. 4, 1812–1821, 10.1002/nop2.826, 33675289. 33675289 PMC8186683

[20] Chang Y. , Hu S. H. , Kuo S. , Chang K. , Kuo C. , Nguyen T. V. , and Chuang Y. , Effects of Board Game Play on Nursing Students′ Medication Knowledge: A Randomized Controlled Trial, Nurse Education in Practice. (2022) 63, 10.1016/j.nepr.2022.103412, 35926260. 35926260

[21] Kotp M. H. , Bassyouny H. A. A. , Aly M. A. , Ibrahim R. K. , Hendy A. , Attia A. S. , Mekdad A. K. , Hafez A. A. , Farghaly Abdelaliem S. M. , Baghdadi N. A. , Hendy A. , and Ismail H. A. , Game on or Game Over? Gamification From 360-Degree Perspective, Perception, Confidence, and Challenges in Simulation Based Nursing Education: Mixed-Method Study, BMC Nursing. (2025) 24, no. 1, 10.1186/s12912-025-03253-z, 40426146. PMC12108018 40426146

[22] Luctkar-Flude M. , Woo K. , Wilson-Keates B. , Larocque M. , Killam L. , Shipton N. , and Tyerman J. , Development of Virtual Simulation Games About Wound Assessment and Management for Nurses and Nursing Students, Teaching and Learning in Nursing. (2025) 20, no. 4, 406–411, 10.1016/j.teln.2025.04.010.

[23] Çelik G. K. , Çalhan K. , Ertural D. , and Odabaşı G. , The Impact of Using the Wound Care Escape Room as a Teaching Game on the Opinions and Motivation of Nursing Students, Journal of Education and Research in Nursing. (2023) 20, no. 4, 318–322, 10.14744/jern.2022.2237.

[24] Sailer M. and Homner L. , The Gamification of Learning: A Meta-Analysis, Educational Psychology Review. (2020) 32, no. 1, 77–112, 10.1007/s10648-019-09498-w, 2-s2.0-85071053037.

[25] Santos A. C. G. , Oliveira W. , Hamari J. , Rodrigues L. , Toda A. M. , Palomino P. T. , and Isotani S. , The Relationship Between User Types and Gamification Designs, User Modeling and User-Adapted Interaction. (2021) 31, no. 5, 907–940, 10.1007/s11257-021-09300-z.

[26] Lee C. , Lee C. , Lai H. , Chen P. , Chen M. , and Yau S. , Emerging Trends in Gamification for Clinical Reasoning Education: A Scoping Review, BMC Medical Education. (2025) 25, no. 1, 10.1186/s12909-025-07044-7, 40133879. PMC11938692 40133879

[27] Seymour A. , Borggren M. , and Baker R. , Escape the Monotony: Gamification Enhances Nursing Education, Journal of Emergency Nursing. (2023) 49, no. 6, 805–810, 10.1016/j.jen.2023.06.004, 37422743. 37422743

[28] John B. and Thomas R. , Gamification as an Innovative Tool in Classroom Teaching: Does It Enhance Learning Outcomes and Student Participation in Nursing?, Journal of Education Technology in Health Sciences. (2024) 10, no. 3, 57–63, 10.18231/j.jeths.2023.014.

[29] Chan S. and Smith G. , Rethinking Gamification for Chinese Nursing Students: A Reflexive Thematic Analysis Study, Nurse Education in Practice. (2025) 83, 104291, 10.1016/j.nepr.2025.104291, 39938129. 39938129

[30] Macalindin B. V. , Ahmed H. F. , Granaghan R. M. , and Goodfellow D. , Improving Nurses′ Digital Literacy and Engagement With Digital Workflows Through a Data-Driven Education Model, Nursing Management. (2024) 31, no. 3, 20–26, 10.7748/nm.2023.e2113, 38014494. 38014494

[31] Nylén-Eriksen M. , Stojiljkovic M. , Lillekroken D. , Lindeflaten K. , Hessevaagbakke E. , Flølo T. N. , and Tørris C. , Games-Thinking; Utilizing Serious Games and Gamification in Nursing Education–A Systematic Review and Meta-Analysis, BMC Medical Education. (2025) 25, no. 1, 10.1186/s12909-024-06531-7. PMC11776282 39881301

